# Chitosan-based nanoparticles as a targeted drug delivery system to treat thyroid cancer: A literature review

**DOI:** 10.1097/MD.0000000000050490

**Published:** 2026-09-04

**Authors:** Hamad S. Alyami, Nora A. Ahmadi

**Affiliations:** a Department of Pharmaceutics, College of Pharmacy, Najran University, Najran, Saudi Arabia; b Health Research Center, Najran University, Najran, Saudi Arabia; c Department of Chemistry, College of Science and Arts, Najran University, Najran, Saudi Arabia; d Creativity in Development School, Najran Education Administration, Najran, Saudi Arabia.

**Keywords:** chitosan-based nanoparticles, drug delivery system, nanomedicine, thyroid cancer

## Abstract

**Background:**

Systemic toxicity, poor drug selectivity, treatment resistance, and inconsistent tumor responses are some of the therapeutic issues related to thyroid cancer (TC). Chitosan-based nanoparticles (ChNPs) have shown promise as nanocarriers for target drug delivery due to their mucoadhesive qualities, biodegradability, biocompatibility, and simplicity of surface modification.

**Methods:**

With an emphasis on their role in enhancing drug delivery and targeting efficiency, this review compiled the literature on recent developments in the design, functionalization, and use of ChNPs for TC therapy.

**Results:**

According to available data, physicochemical changes such as ligand conjugation, PEGylation, and stimuli-responsive linkers can support the stability, cellular uptake, circulation time, and therapeutic efficacy of NPs. ChNPs can deliver anticancer drugs to thyroid carcinoma cells with greater selectivity by utilizing both active targeting through ligand-mediated interactions and passive targeting through the increased permeability and retention effect. When compared to traditional therapeutic methods, preclinical research has shown better boosted antitumor activity, medication absorption, and dimensioned systemic toxicity. However, difficulties with large-scale production, regulatory approval, and long-term safety evaluation continue to hinder clinical translation.

**Conclusion:**

Although further translational and clinical research is required to confirm ChNPs’ safety and efficacy in standard clinical practice, they offer a promising platform for targeted TC treatment.

## 1. Introduction

### 1.1. Structure and function of the thyroid gland

**Figure 1.**
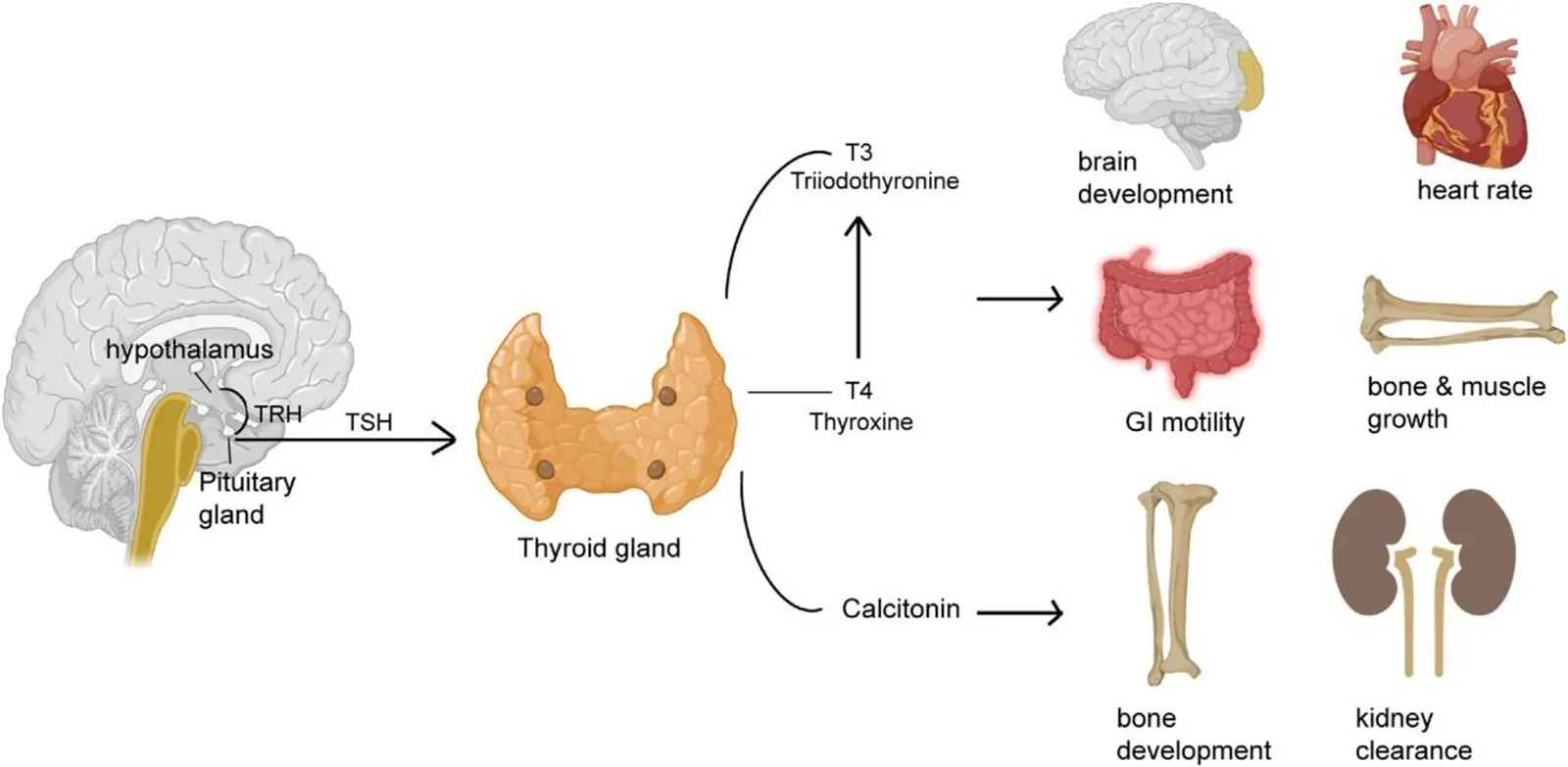
Thyroid gland anatomy and physiological function. TRH = thyrotropin-releasing hormone, TSH = thyroid-stimulating hormone.

The thyroid gland, “a butterfly-shaped endocrine organ in the anterior neck,” is responsible for the synthesis and secretion of the triiodothyronine (T3) and thyroid hormones thyroxine (T4). Both T3 and T4 hormones are important for physiological processes in the body, including growth and metabolism. The anatomical structure of the thyroid gland is demonstrated in Figure 1.^[1]^

### 1.2. Thyroid cancer: epidemiology, incidence, and classification

**Figure 2.**
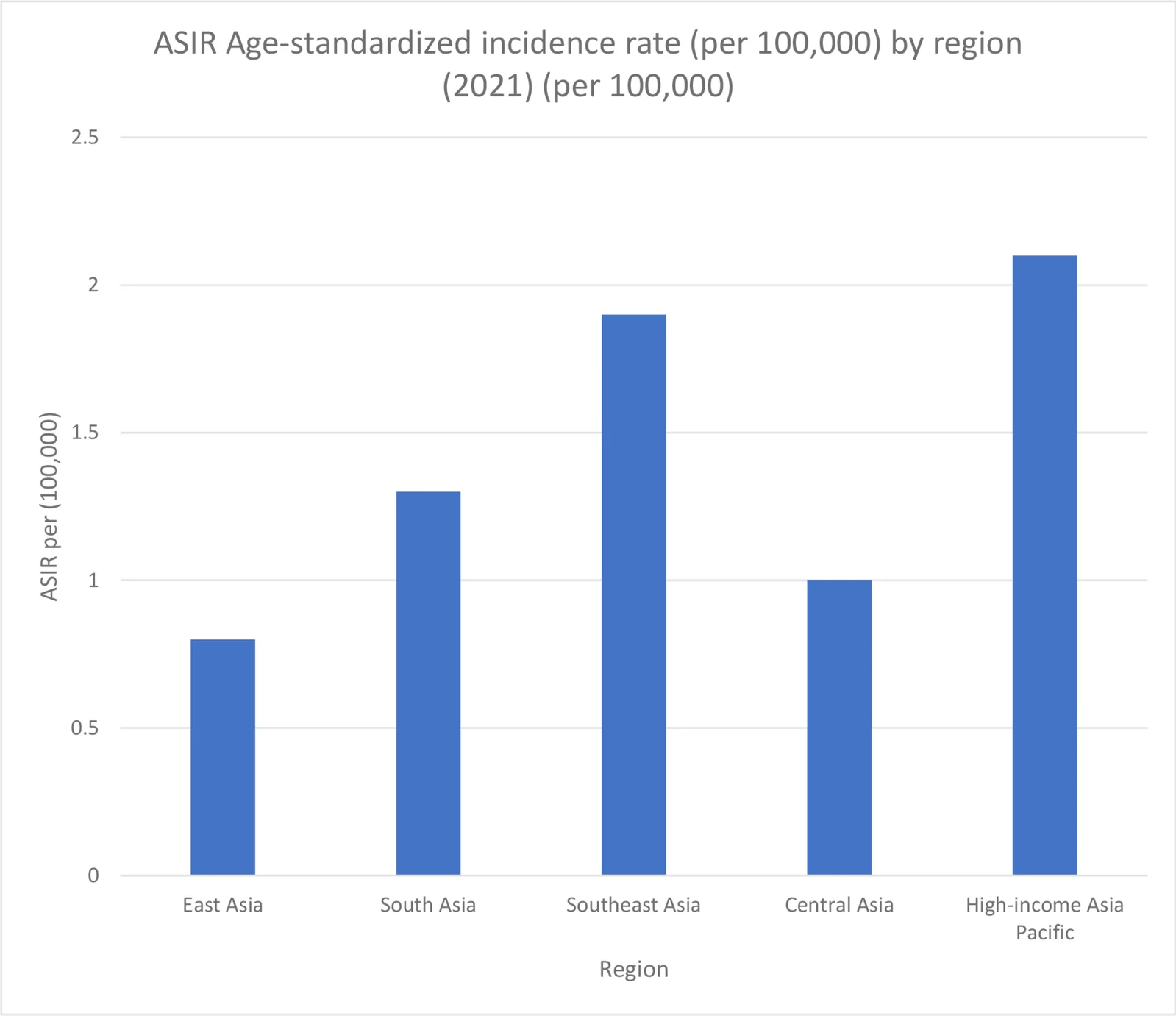
Regional trends of thyroid cancer in Asia. ASIR = age-standardized incidence rate.

**Figure 3.**
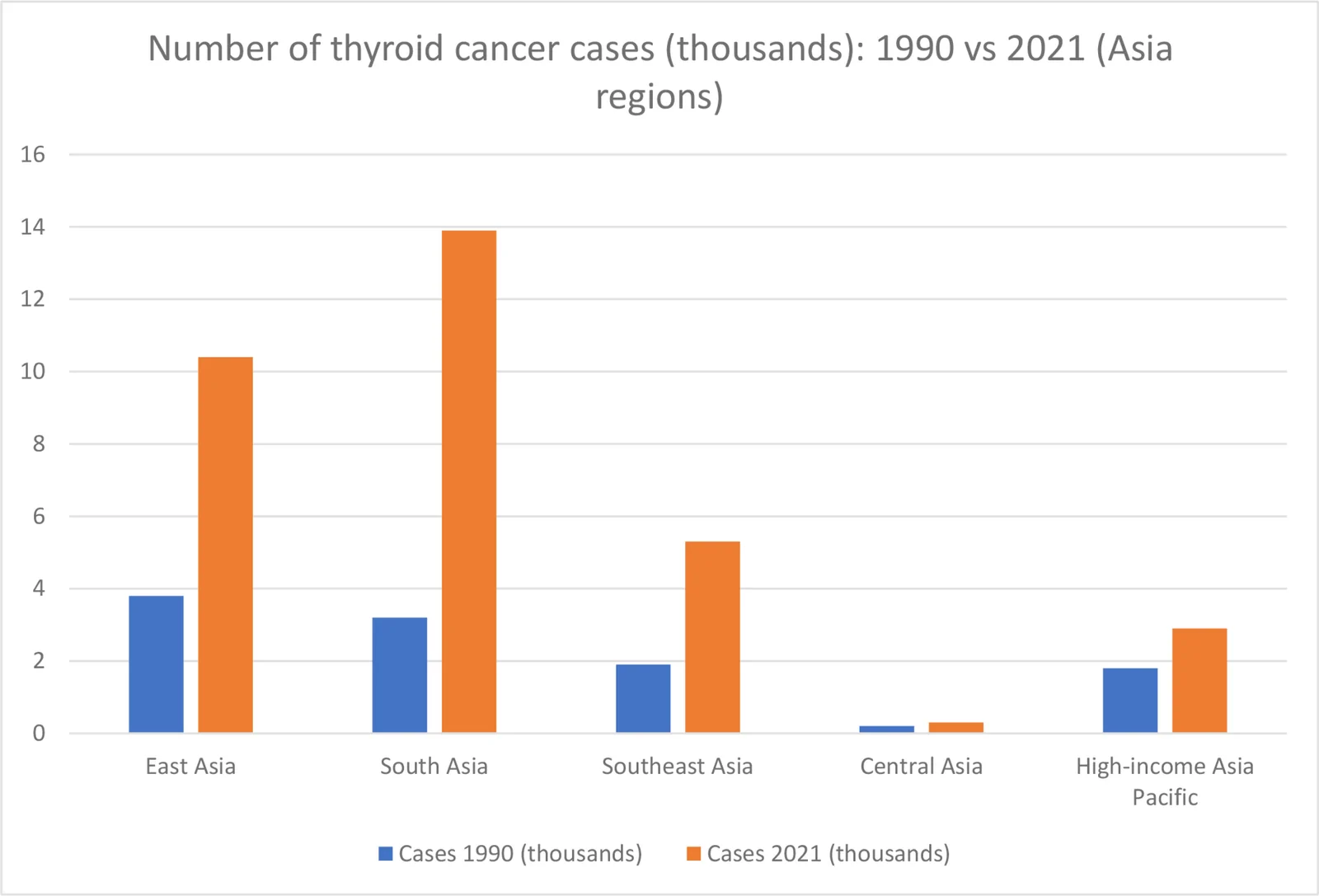
The change in total cases from 1990 to 2021.

In 2022, thyroid cancer (TC) was the most prevalent endocrine system cancer worldwide, with an estimated 821,214 new cases and 47,507 deaths globally.^[2]^ Although TC accounts for approximately 4% of all cancer diagnoses, its incidence and burden continue to rise, particularly in regions with high human development.^[3]^ The age-standardized incidence rate has increased by 1.25% annually to 2.91 per 100,000. The overall burden is still considerable, even though mortality has decreased. The highest global incidence rate was recorded by Saudi Arabia, underscoring regional differences.^[4]^ In 2022, Asia accounted for 72.7% of global TC cases and 61.3% of mortality due to TC, reflecting both the highest incidence rate of TC globally and the highest number of TC cases.^[5]^ Figures 2 and 3 present regional trends of TC in Asia (1990–2021) and the number of TC cases. They show a rising incidence in South and East Asia, stability in Central Asia, and modest growth in the high-income Asia Pacific region.

### 1.3. Therapeutic challenges in thyroid malignancies

Due mostly to the growing use of neck ultrasonography and fine needle aspiration biopsy, thyroid surgery has become one of the most used surgical operations, while the incidence of TC has significantly increased globally over the past 10 years. More tumor instances, especially microcarcinomas, have been detected in recent years thanks to the exponential expansion of these procedures.^[6]^ Complete surgical excision is not usually possible, even though it is the main treatment for differentiated thyroid cancer (DTC). For patients who are at high risk of recurrence, radioactive iodine (RAI) is frequently utilized as an adjuvant; however, its application is restricted in cases of substantial residual or unresectable disease.^[7]^ Chemotherapy, radiotherapy, and surgical resection are the main clinical treatments used today for TC and other cancers. However, there are frequently unavoidable side effects associated with these popular cancer treatments. For instance, in surgical resection, a portion of normal tissue may be removed when the tumor tissues are removed. Chemotherapy can successfully destroy tumor cells and stop them from growing, but it can also weaken the immune system and result in side effects, including nausea and vomiting.^[8]^ Some challenges associated with failure of conventional therapy may occur, such as tumor recurrence, off-target toxicity, and poor medication permeability.

Certain clinical and pathologic features, such as extrathyroidal expansion, aggressive cancer histologies, vascular invasion, regional spread, or increasing postoperative serum thyroglobulin levels suggestive of distant spread, can be used to evaluate the possibility of papillary thyroid carcinoma (PTC) recurrence.^[9]^ Until recently, patients with late-stage TC and incurable local or metastatic disease had restricted access to treatment options, including radiotherapy, chemotherapy, or local therapies.^[10]^ The table below presents factors that influence TC results, such as age or the coexistence of other diseases. The results are presented as a hazard ratios and a corresponding 95% confidence interval with a *P* value.

Table 1 presents a comprehensive analysis of key clinical and pathological variables that affect TC results, emphasizing both univariate and multivariate hazard ratios with 95% confidence interval and *P* values. Adapted from^[11]^; other factors influencing TC outcome will be explained throughout the text.

**Table 1:** Clinical and pathological variables that affect TC results.

Factor	Multivariate HR (95% CI)	*P* value
Age ≥55 yr	2.67 (1.27–5.61)	.010
Coexistence of Hashimoto thyroiditis	0.16 (0.04–0.68)	.013
Multifocality	(0.78–2.98) 1.52	.219

CI = confidence interval, HR = hazard ratio, TC = thyroid cancer.

**Figure 4.**
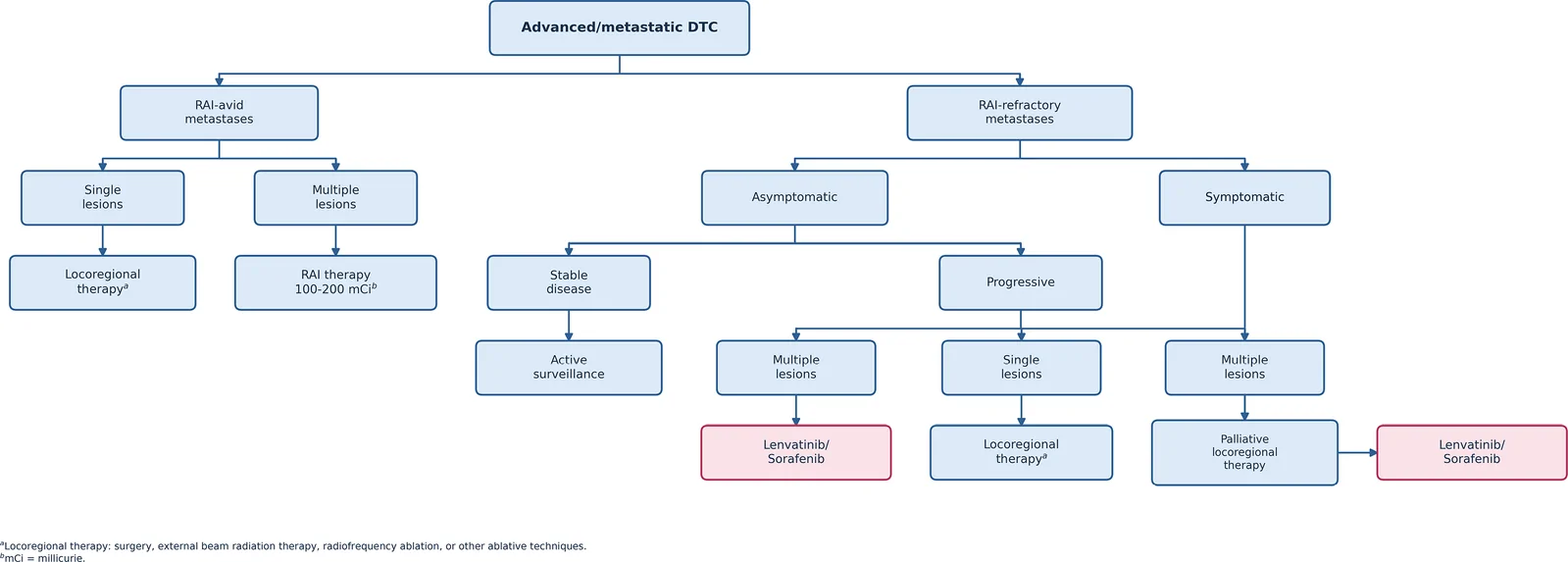
The treatment approach in advanced/metastatic differentiated thyroid cancer. DTC = differentiated thyroid cancer, RAI = radioactive iodine.

Figure 4 presents the treatment approach in advanced/metastatic DTC.^[12]^

### 1.4. Limitations of conventional chemotherapy and drug delivery systems

The most significant obstacle associated with TC therapy is resistance to conventional and gene-specific therapies. Two main obstructing mechanisms have been recognized: mutations in on-target genes that decrease the efficacy of therapy, and the stimulation of bypass signaling pathways, particularly kinase pathways, maintaining tumor progression. For instance, in patients treated with rearranged during transfection (RET)-specific inhibitors, the resistance mechanisms remain poorly characterized, emphasizing the need for more molecular and clinical profiling.^[13]^ In papillary thyroid microcarcinomas (PTMC), due to the slow proliferation rate of PTMCs, the practice of active surveillance and sustained surgery has become an alternative to immediate thyroidectomy. However, a subset of PTMCs exhibit early lymph node spreading and aggressive behavior, necessitating timely surgical intervention.

However, these 2 multi-kinase inhibitors frequently cause off-target side effects that limit their use in patients with RET-driven cancers, including RET-mutated medullary thyroid carcinoma (MTC). The same can be said regarding their utility for patients with RET-related malignancies, such as RET-mutated MTC.^[14]^ Results may be enhanced by reoperation, embolization, and radiation. Meanwhile, conventional chemotherapy has a limited response and can be fatal. Currently, alternatives for treatment are limited and few.^[15]^ There is currently no treatment to cure anaplastic thyroid carcinoma (ATC) patients or increase survival rates. Therefore, the development of innovative therapeutic approaches to combat ATC is urgently needed.^[16]^ Tyrosine kinase inhibitors (such as sorafenib and lenvatinib), inhibitors of the mitogen-activated protein kinase pathway (such as dabrafenib and trametinib), RAI therapy, immune checkpoint inhibitors (such as pembrolizumab and nivolumab), and gene-targeted medicines are among the targeted treatments for TC. These treatments improve the immune system, break down tumor-specific pathways, or eradicate any remaining illness. Although they work well, their use necessitates cautious patient selection and close observation to balance effectiveness and reduce side effects.^[17]^

By facilitating the targeted distribution of medications, genes, and thermal or photodynamic agents, nanoparticles (NPs) improve the treatment of cancer. They also play a role in biomarker identification and imaging. NPs can be hybrid, inorganic, or organic, and their ability to co-deliver enhances the accuracy of treatment. Nanotechnology seeks to minimize systemic toxicity and maximize tumor-specific delivery.^[18]^ Because of their special physicochemical characteristics, such as stability, surface modifiability, and small size, NPs provide targeted drug administration and improved tumor imaging. They can be functional for both active and passive targeting. Drug loading, cellular uptake, and stability are all impacted by NP size. While smaller NPs may assemble, larger NPs transport more medication and penetrate tissues more effectively. Poly (lactic-co-glycolic acid) or chitosan are examples of biocompatible shells used in polymeric nanocapsules (100–500 nm). Because of its high binding affinity and focused activity, chitosan has great antibacterial delivery potential.^[19]^ In light of off-target toxicities, resistance mechanisms, and restricted curative options that are associated with conventional chemotherapy and targeted agents, alternative drug delivery approaches, particularly chitosan-based nanoparticle (ChNP) systems, have garnered a growing amount of attention for their potential to improve therapeutic efficacy and reduce systemic toxicity in comparison to conventional treatment. However, important translational challenges remain before their routine clinical implementation can be achieved.

### 1.5. Emergence of nanomedicine in cancer therapy

The field of medicine that uses nanotechnology for disease prevention, monitoring, and intervention through novel imaging, diagnostic, therapy, repair, and regeneration techniques for biological systems is known as nanomedicine. Nanomedicine research aims to enter the clinic and enhance the health or quality of life of the patient, whether this is through the development of more effective biomaterials for tissue regeneration, novel (or improved) medicines, or diagnostic techniques.^[20]^ To improve efficacy and combat medication resistance, 2nd- and 3rd-generation nanomedicines seek to precisely target tumors, 1st at the tissue and cellular level using ligands (such as antibodies or peptides), and later at the organelle level (such as the nucleus or mitochondria). Specificity is increased by biomimetic techniques employing NPs coated in cell membranes. Stimuli-responsive nanocarriers are being designed to dynamically adjust characteristics (size, charge, and ligand exposure) in response to conflicting requirements across targeting phases. Despite their great therapeutic promise, these hierarchical systems continue to encounter translational hurdles.^[21]^ To improve tumor selectivity, cancer nanomedicines employ both passive (the enhanced permeability and retention [EPR] effect) and active (ligand-based) targeting. However, recent studies have questioned the clinical relevance of the enhanced EPR effect, as its efficacy appears limited and highly variable in human tumors compared to models. Consequently, researchers are increasingly focusing on strategies that combine EPR-based passive accumulation with active or stimuli-responsive targeting to enhance tumor delivery efficacy.^[22,23]^ As Figure 4 illustrates, these drug delivery strategies are mediated by NPs, including active ligand-based targeting and passive accumulation via the EPR effect.

**Figure 5.**
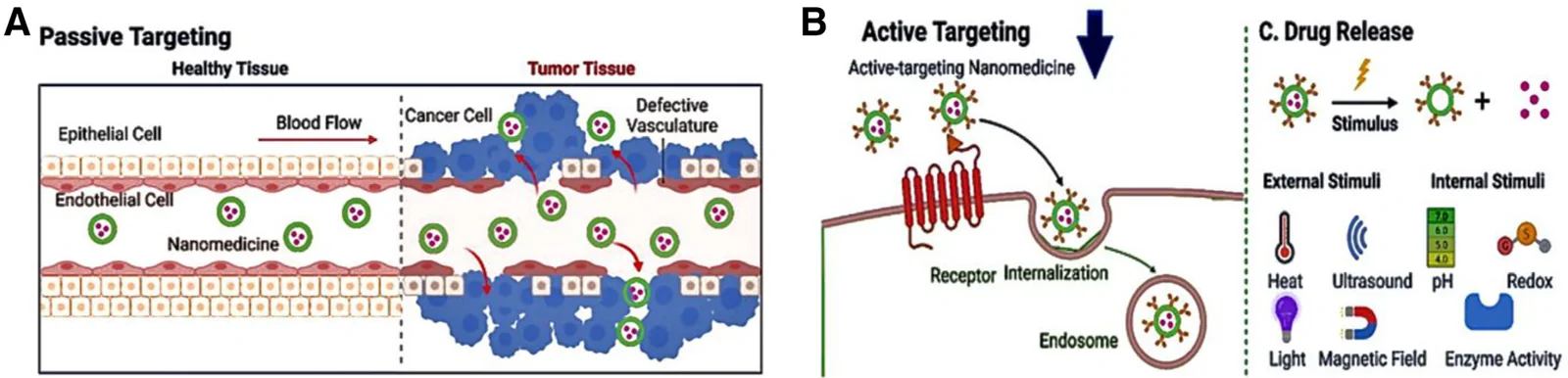
Mechanisms of nanoparticle-mediated drug delivery. Panel A shows passive targeting via the EPR effect, Panel B shows active targeting via receptor-mediated endocytosis, and Panel C shows stimuli-responsive drug release.

Figure 5 presents the adapted and modified mechanisms of NP-mediated drug delivery.^[24]^ A schematic illustration shows how NPs deliver therapeutic agents to tumor cells. The figure highlights major mechanisms, including passive targeting through the enhanced EPR effect, active targeting via ligand-receptor interactions, cellular uptake by endocytosis, intracellular drug release, and accumulation at the tumor site.

A common chemotherapeutic treatment for solid tumors, paclitaxel has issues with low solubility, poor stability, and toxicity from solvents. Its pharmacokinetic profile and therapeutic performance have been enhanced by formulations based on nanotechnology, allowing for targeted distribution with fewer adverse effects. Research is continuing to improve the safety and tumor-specific efficacy of paclitaxel NPs, which are now approved for the clinical treatment of lung, ovarian, and breast malignancies.^[25]^ With a progression-free survival of >3 months, ATC responds poorly to conventional chemotherapy because of chemoresistance and severe toxicity. The sole licensed medication, doxorubicin (DOX), is not very effective. The delivery of DOX in resistant ATC cells is improved by NPs, particularly bovine serum albumin (BSA)-stabilized DOX-loaded mesoporous organosilica, which increase drug uptake and decrease efflux.^[26]^ The distinct physicochemical characteristics of nanomedicines, including liposomes, poly(lactic-co-glycolic acid) (PLGA) NPs, dendrimers, and metal oxides (e.g., FeO_4_, GdO_3_, and MnO_4_), such as their small size, large surface area, modifiable surfaces, magnetic behavior, and high biocompatibility, allow for precise immune targeting and disease imaging.^[27]^ This review aims to provide a comprehensive and current overview of ChNPs as targeted drug delivery systems (DDSs) for TC. Unlike previous reviews that have broadly discussed nanomedicine or NP-based cancer therapies, the present review specifically focuses on the unique physicochemical properties of chitosan, surface-engineering strategies, targeting mechanisms, drug-loading characteristics, and translational challenges associated with ChNPs in TC management. A special focus is on how ChNPs might overcome some of the main drawbacks of traditional treatments, such as limited tumor-specific accumulation, poor systemic toxicity, drug selectivity, and treatment resistance. Furthermore, the present barriers to clinical translation, such as regulatory concerns, manufacturing scalability, and long-term safety evaluation, are also critically examined in this review. This review offers a focused viewpoint that may encourage further research and assist in the development of more potent targeted therapy approaches by fusing recent developments in nanotechnology with the clinical difficulties of treating TC.

### 1.6. Search strategy and literature selection

An extensive literature review was conducted using multiple electronic databases, such as Scopus, PubMed, and Google Scholar. Articles that were published prior to 2026 were the primary focus of the search strategy. We implemented the following keywords and Boolean search combinations: “chitosan nanoparticles AND cancer therapy,” “chitosan nanoparticles AND drug delivery,” “thyroid cancer AND nanomedicine,” “thyroid cancer AND nanoparticles,” “chitosan AND targeted drug delivery AND cancer,” “tumor microenvironment AND nanoparticles,” “anaplastic thyroid cancer AND nanoparticles,” “EPR effect AND nanoparticles,” and “thyroid cancer AND global burden.” Studies were included if they were original research articles, systematic reviews, or narrative reviews published in English that addressed the synthesis, physicochemical characterization, drug loading and release, or therapeutic application of chitosan NPs in cancer, with a specific focus on TC. Otherwise, the studies were excluded if they were non-English publications, conference abstracts, editorials, or studies that had no direct association with chitosan-based nanomedicine or thyroid pathology.

## 2. Chitosan: chemistry, properties, and biomedical significance

### 2.1. Molecular structure and sources of chitosan

Chitosan, a biodegradable polymer obtained from chitin, possesses unique physicochemical and biological properties such as biocompatibility, positive charge, and activity against bacteria, which make it suitable for DDS applications.^[28–31]^

Green and enzymatic processes are environmentally friendly strategies for the production of chitosan. Biological extraction is done by demineralization and deproteinization, fermentation with Lactobacillus or fungi, while enzymatic deacetylation makes use of chitin deacetylase. Despite allowing cleaner products with reduced environmental impact, these methods are restricted in terms of cost and scalability.^[32]^

The effectiveness of both chitin and chitosan as adsorbent materials arises from their chemical structure, which contains amine groups at the C2 position of glucosamine residues, hydroxyl groups on both glucosamine and *N*-acetylglucosamine units, and methyl-containing acetyl groups within *N*-acetylglucosamine. Because of deacetylation, chitosan contains more –NH_2_ groups and fewer –CH_3_ groups than chitin. Since the –OH concentration of both polymers is identical, the –NH_2_ and –CH_3_ groups are primarily responsible for the variations in adsorption behavior.

### 2.2. Chemical derivatization and functionalization of chitosan (e.g., glycol chitosan and carboxymethyl chitosan [CMCS])

A promising option for drug delivery applications, glycol chitosan is a water-soluble derivative of chitosan that has exceptional biocompatibility and biodegradability. Its solubility in aqueous fluids is greatly increased by the presence of glycol groups, which makes it easier to utilize in polymeric drug carriers. Furthermore, glycol chitosan has shown promise in creating hydrogels that can self-heal after damage. Because of their adjustable mechanical and healing properties, these hydrogels have attracted interest for controlled medication release and cell encapsulation.^[33,34]^ As previously mentioned, the loading of ChNPs with drug molecules and deoxyribonucleic acid is made possible by the presence of the amino and hydroxyl functional groups as well as the glycosidic link. Another benefit of employing ChNPs is that they enable the controlled release of medications in vivo through mucoadhesion. ChNPs have several significant potential uses.^[31]^

**Figure 6.**
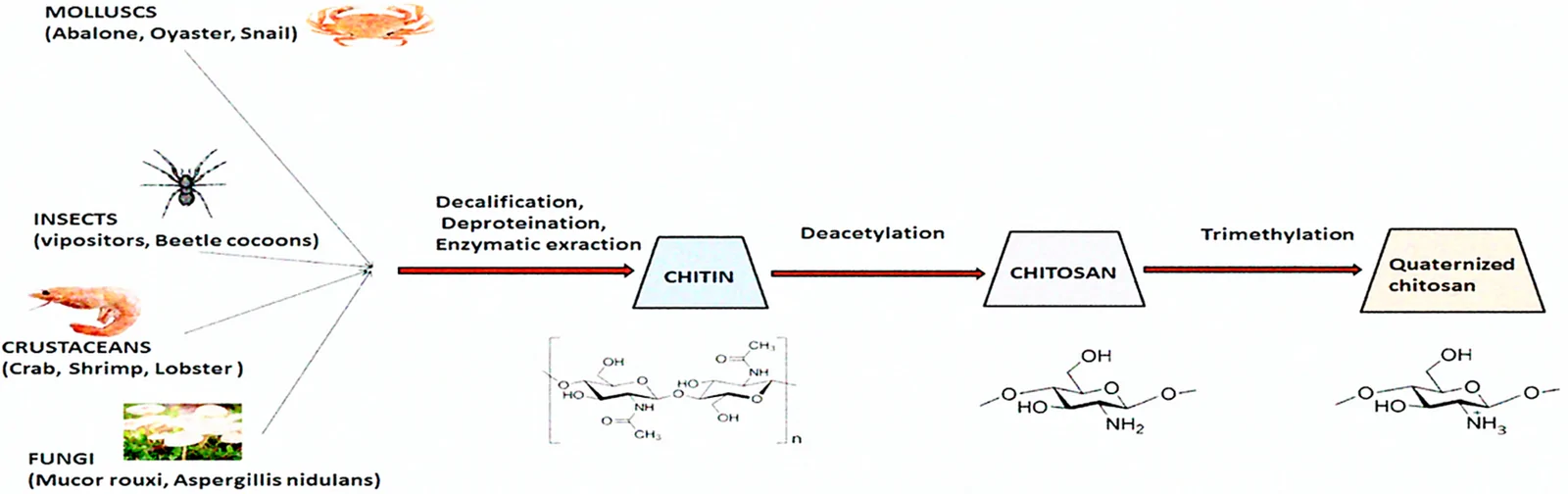
Transformation process from chitin to chitosan and its derivatives.

Figure 6 schematically presents the transformation from chitin to chitosan. Adapted from^[35]^ the figure showed the chemical deacetylation, a chemical modification to generate functional derivatives.

Glycol chitosan (GC) NPs enable controlled release and efficient drug encapsulation, which is reflected in improved medication pharmacokinetics. Based on a drug’s properties and formulation techniques, drugs can be entrapped either during the combination of NPs or incubation. Collectively, water absorption, osmotic pressure, matrix penetration, erosion, and other diffusion and polymer breakdown processes control drug release. Because GC is biodegradable, its release profiles may be adjusted, enhancing medication stability, prolonged administration, and bioavailability – all of which are essential for the effectiveness of cancer treatment.^[36]^ Injectable, self-healing, pH-responsive hydrogels with a high drug-loading capacity can be created thanks to CMCS (Quaternized Chitosan). The efficient loading and release of acetylsalicylic acid by these hydrogels, which were created by dynamic imine and hydrazone linkages with oxidized hyaluronic acid (HA) and adipic dihydrazide, support their application in intelligent DDSs.^[37]^ Furthermore, several medications or therapeutic agents with complementary modes of action can be delivered simultaneously because of the versatility of nanomedicines and nanocarriers. Drug resistance can be overcome, treatment efficacy increased, and the chance of tumor recurrence decreased using this coordinated approach. The main innovative approaches now depend on the use of nanomedicines and nanocarriers and should be extended for biomolecule transportation.^[38]^ By increasing chitosan’s solubility and drug-loading ability, carboxymethylation makes it a potentially effective delivery system for anticancer drugs such as DOX. The 2 issues that are highly associated with conventional chemotherapy are high systemic toxicity and multidrug resistance. By loading DOX, CMCS enhances intracellular drug concentration, decreases side effects, and allows regulated release, all of which contribute to improved therapeutic efficacy.^[39]^

### 2.3. Key physicochemical properties of chitosan relevant to drug delivery

The physiological characteristics of chitosan, such as its molecular weight (MW) and the degree of deacetylation (DD), affect its solubility, stability, and interaction with drugs, thus influencing NP creation.^[40,41]^ The viscosity of the chitosan solution and the mechanical entanglement of polymer chains during ionic gelation are primarily influenced by the MW. Low-MW chitosan yields smaller particles with weaker mechanical stability and reduced polydispersity index. High-MW chitosan produces larger, more polydisperse NPs with slower degradation. Chitosan with a lower MW generates DDSs with smaller particle sizes and lower polydispersity.^[42]^ In contrast, the DD directly influences the zeta potential of the resulting NPs and their ability to electrostatically cross-link with anionic agents like tripolyphosphate (TPP). While higher DD enhances colloidal stability and cellular uptake, it also reduces biodegradability; lower DD reduces uptake efficiency and zeta potential NP uptake. In order to optimize particle size, encapsulation efficiency, and drug release kinetics, MW and DD must be well-balanced.^[42]^

### 2.4. Biocompatibility, biodegradability, and mucoadhesive properties of chitosan

**Figure 7.**
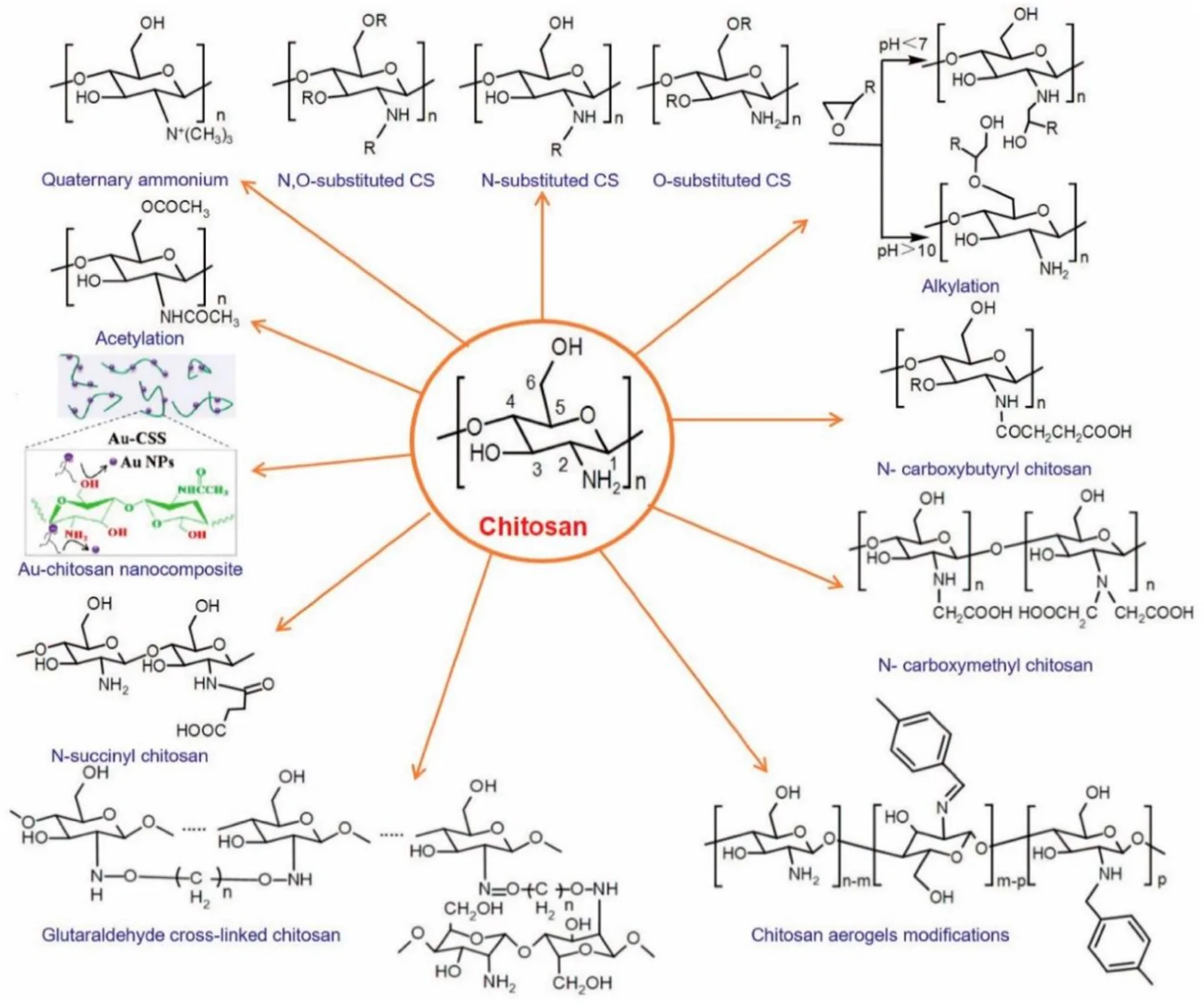
Chemical structure of different types of chemical modification of chitosan.

A common polysaccharide in biomedical applications, chitosan is nontoxic, biocompatible, and biodegradable. Enzymes like lysozyme can break it down naturally in the body, and its hemostatic and wound-healing qualities are associated with interactions with platelets. Its cationic properties enhance its function as an immunological adjuvant and drug carrier, particularly in tissue engineering, gene transfer, and cancer treatments, Figure 7.^[43]^

## 3. Fabrication and engineering of ChNPs

### 3.1. Fabrication methods: ionic gelation, emulsification, spray drying

Experimental factors such as the chitosan/TPP ratio, chitosan MW, pH, and stirring parameters have a significant impact on the physicochemical properties of the resultant NPs, including size, surface charge, and polydispersity. Ionic gelation remains a fundamental method for creating ChNPs because of its simplicity, reproducibility, and versatility.^[44]^ NP physicochemical properties, such as size, surface charge, and homogeneity, can be influenced by the chitosan/TPP ratio, chitosan MW, pH, and stirring settings. Another popular method for making ChNPs is emulsification. The process creates emulsion droplets, or reverse micelles, that function as nanoreactors by combining an acidic chitosan solution with an oil phase that contains surfactants. Controlled NP production with enhanced size homogeneity and stability is made possible by this constrained environment. The resultant NPs can be utilized for a variety of drug delivery applications after centrifugation, washing, and drying.^[45,46]^

One common 1-step process for creating dry micro-NPs is spray drying. It permits temperature control during processing and does away with the requirement for organic solvents. Although spray-dried particles usually have a uniform shape and strong thermal stability, processing can cause the high viscosity of the chitosan solution and particles to agglomerate, which can lead to fast drug release and particle size variability.^[47]^ Process parameters and agents such as TPP can overcome these limitations and can improve the biocompatible NPs appropriate for targeted drug delivery.^[48,49]^

Because microfluidic technologies can precisely control the creation of NPs at the microscale, they have become a particularly effective strategy. Various flow regimes, including hydrodynamic focusing and vortex creation, can be created by adjusting flow rates in microfluidic platforms. This allows for controlled NP formation by solvent displacement in X-shaped microdevices. For use in biomedical applications, this produces NPs with a restricted size distribution, great repeatability, and enhanced scalability. Rapid mixing and consistent processing conditions are provided by microfluidic systems, resulting in monodisperse ChNPs with precisely controlled physicochemical characteristics.^[50,51]^

ChNPs may exhibit poor physicochemical stability in aqueous media, leading to aggregation and fusion during long-term storage. Lyophilization (freeze-drying) is widely used to improve their stability by preserving particle integrity and preventing aggregation, while also enhancing long-term storage and redispersibility. However, in the absence of cryoprotectants, freeze-dried NPs may agglomerate upon reconstitution. Therefore, stabilizing agents such as trehalose, mannitol, sorbitol, or glycerol are essential to protect NP structure and maintain performance. Although lyophilization improves storage stability, the process can induce stress due to dehydration and strong intermolecular interactions, potentially altering properties such as zeta potential, viscosity, and water uptake, which may also affect drug release behavior. Careful optimization of formulation and cryoprotectant selection is, therefore, required to ensure NP stability and functionality.^[52,53]^ Table 2 presents ChNP fabrication methods and their main characteristics.^[54]^

**Table 2:** Comparative overview of fabrication methods for ChNPs: physicochemical properties, advantages, and applications.

Methods	Process	Advantages	Disadvantages
Ionic gelation	Ionic cross-linking is activated by mixing an aqueous solution containing chitosan and another containing TPP, thus resulting in a complex coacervate aqueous phase	Straightforward procedure using mild chemicals. NP size is easily regulated by altering the concentration of chitosan and TPP	Difficult to produce uniformly sized NPs.
Microemulsion/reverse micelle	Based on covalent cross-linking where a reverse micelle is formed, introducing a surfactant into an organic solvent and then adding the mixture to an appropriate acidic solution containing chitosan.	A straightforward procedure achieving greater uniformity of the size of NPs.	Use of harmful chemicals and a time-intensive process.
Emulsification solvent diffusion method	Polymeric precipitation resulting in the formation of nanoparticles	Straightforward procedure.	Substantial shear forces occur during the formation of ChNPs.
Polyelectrolyte complex method	A self-assembly occurs due to the electrostatic interaction between the oppositely charged chitosan and the added polymer or counter ion, resulting in charge. neutralization.	NP size can be regulated by pH of the solution, molecular weight (MW), and concentration of the constituents.	Due to the neutralization of charge, the PEC is self-assembled, leading to a substantial reduction in hydrophilicity.

ChNPs = chitosan-based nanoparticles, NP = nanoparticle, PEC = polyelectrolyte complex, TPP = tripolyphosphate.

### 3.2. Particle size, zeta potential, and morphological considerations

The biological efficacy of ChNPs is highly dependent on their physical properties. Drug distribution, permeability, and cellular absorption are all impacted by particle size, while surface charge affects the zeta potential, thus affecting colloidal stability and aggregation. In addition, morphology influences drug release pattern, density, and surface area. Physicochemical and functional properties are characterized by dynamic light scattering, transmission electron microscopy, and scanning electron microscopy (SEM).^[55,56]^

The surface charge and colloidal stability are identified by the protonation state of amino groups; pH has a role in the zeta potential of ChNPs. The best conditions to improve electrostatic repulsion and inhibit aggregation are high positive charges and low pH. A reduction in protonation makes the zeta potential low at neutral or alkaline pH, thus causing a decrease in stability and particle agglomeration. As its stability has a direct impact on functionality and bioavailability, this pH-dependent behavior is essential for food packaging applications and DDSs.^[57]^ Significant electrostatic interactions between the positive charges of NPs and the negative charges of cellular membranes permit cellular uptake and adhesion. Moreover, suitable surface charges promote localized distribution of the drug, sustain systemic circulation, and minimize particle aggregation. This is critical in antitumor implementations, where zeta potential is crucial to enhance therapeutic effectiveness at the target site and decrease premature drug release in nontarget tissues.^[58]^

### 3.3. Surface functionalization for targeting: ligands, antibodies, and targeting moieties

ChNPs with altered surfaces are more stable, without aggregation, and interact with biological barriers more effectively. These impact toxicity, targeted drug administration, circulation, drug release, and cellular uptake by cancerous cells. Surface engineering is critical for improving nanotechnology because it allows NPs to achieve high levels of therapeutic efficacy and bioavailability via adjusting their surface charge, hydrophilicity, and functional groups.^[59]^ Some factors, such as quick elimination from circulation as a result of opsonization, protein corona formation, and aggregation, can limit the targeting efficacy of NPs. Some technologies, such as PEGylation, protect NPs to improve biodistribution, stability, and circulation duration. In DDSs, this “stealth” approach optimizes therapeutic efficacy, controlled drug release, and specificity.^[60]^ PEGylation helps improve tumor drug accumulation, circulation time, and NP stability. ChNPs also enhance drug delivery, intracellular uptake, and clinical efficacy, making them appropriate for targeted cancer therapy. Controlled PEGylation strikes a compromise between effective cellular absorption and extended systemic availability.^[61]^

Biological ligands increase cancer cell absorption of NPs while minimizing off-target effects. Important ligands include antibodies specific for tumor antigens; aptamers, which are small and highly specific deoxyribonucleic acid/ribonucleic acid ligands; folic acid, which targets folate receptors; HA, which targets CD44, and transferrin, which targets transferrin receptors. These ligands are important for active targeting applications in nanomedicine because they increase the selectivity, intracellular delivery, and therapeutic efficacy of the tumor.^[60]^ Biological ligand conjugation, which improves cell uptake via receptor-mediated endocytosis, and functionalizing NPs with ligands (such as folate, transferrin, HA, antibodies, or aptamers), allow selective binding to overexpressed cancer receptors. By comparison, using non-covalent connections, which impact circulation, bioavailability, and tumor accumulation, is less stable but simpler than using covalent interactions, which offer stable attachment.^[62]^

ChNPs can be used to transport drugs to cancer or brain tumors precisely and effectively. They prevent many challenges regarding multidrug resistance, lower the risk of systemic toxicity, and improve drug concentration in tumors by combining active ligand-mediated targeting with passive targeting (the EPR impact). Multifunctional ChNPs are effective equipment for accurate treatment because they can also deliver imaging agents and modify the tumor microenvironment.^[35]^ Off-target toxicity, a fall in drug absorption, and obstacles like the blood–brain barrier are some of the difficulties facing cancer treatment. By increasing medication concentrations at targeted tissues and cells and minimizing off-target toxicity, nanocarriers, specifically polysaccharide-based NPs, can be effective. Due to their low immunogenicity, their receptiveness to chemical modification, biocompatibility, and biodegradability, natural polymers such as pectin and chitosan are appropriate for forming nanoscale carriers that can cross the blood–brain barrier and deliver doses of therapeutic drugs to tumor cells. Furthermore, these systems can impact drug stability, drug release, bypass efflux mechanisms, and combination therapy, enhancing the efficacy against both breast and brain tumors.^[63]^

### 3.4. Encapsulation efficiency and loading capacity of ChNPs

Loading capacity (LC%) is the ratio of the loading drug to the total weight of the NP. Encapsulation efficiency (EE%) is the percentage of the drug that is successfully encapsulated inside the NP matrix in relation to the total amount of drug used. The efficacy of the preparation procedure and the ability of ChNPs to serve as good drug vehicles are both directly reflected in these properties, making them important physicochemical parameters.^[64]^ When evaluating delivery systems based on NPs, EE% and LC% must be considered. While ideal LC shows the system’s capability to carry a significant dose without using extra carrier material, high EE guarantees that a sizable fraction of the chemotherapeutic agent is entrapped within the nanocarrier. These factors make ChNPs attractive options for innovative cancer therapy since they affect drug release pharmacokinetics, stability, therapeutic efficacy, and the decrease of systemic toxicity.^[41]^

A drug’s LC and EE are highly influenced by the NP production technique. Some techniques, such as flash nanoprecipitation, which rapidly mixes polymer and drug solutions, improve EE and LC by enabling homogeneous nucleation and tiny particle sizes. Temperature is a vital factor in NP preparation. While elevated temperatures can increase a drug’s solubility and diffusion, facilitating drug loading, they also risk inducing drug degradation and compromising NP stability. Therefore, temperature and preparation technique must be carefully optimized to ensure adequate EE and LC while maintaining formulation stability.^[65]^ During synthesis, low temperatures, ranging from 10°C to 25°C, are used to reduce squalene breakdown and stop particle aggregation. While keeping the NPs at 4°C in the dark maintains the bioactivity of squalene and efficient EE, ice-bath sonication guarantees consistent particle size and improved stability.^[66]^

### 3.5. Stability, drug release kinetics, and shelf-life considerations

To prevent the gathering and loss of medication, maintaining the stability of ChNPs is critical. Sometimes, chitosan can cause oxidation, depolymerization, and polymer degradation because of its hygroscopic nature and susceptibility to environmental conditions, including temperature and humidity, which will, in turn, change its physicochemical characteristics.^[67]^

## 4. Targeting strategies and mechanisms in TC therapy

### 4.1. The tumor microenvironment of TC and its implications for nanomedicine^[68,69]^

Based on cellular origin and differentiation, TC can exhibit a variety of behaviors. Well-differentiated (DTCs: papillary, follicular, and oncocytic) subtypes have a good prognosis, while poorly differentiated (poorly differentiated thyroid carcinoma/differentiated high-grade thyroid carcinoma) and ATC subtypes are highly invasive and metastatic. Histology, mutational status, and signaling pathways – particularly mitogen-activated protein kinase (Rat Sarcoma [RAS]–rapidly accelerated fibrosarcoma–mitogen-activated protein kinase–extracellular signal-regulated kinase and phosphoinositide 3-kinase/protein kinase B), which control proliferation, apoptosis, and metastasis – all affect tumor aggressiveness and microenvironmental interactions. Important mutations, such as RAS isoforms and BRAFV600E, influence the immune system, tumor growth, and therapeutic response, underscoring their vital significance in targeted and immunotherapeutic approaches.^[70]^ The tumor microenvironment (TME) strongly influences the effectiveness of immunotherapy. Tumor-associated macrophages, immunological checkpoint proteins, such as programmed cell death protein 1 and cytotoxic T-lymphocyte-associated protein 4, and stromal cells are some components of the TME that influence antitumor immune responses and maintain immunosuppressive environments, particularly in aggressive subtypes like ATC. Tumor immunogenicity is influenced by the burden of gene mutations, such as B-rapidly accelerated fibrosarcoma and RAS, which affects the responsiveness to immune checkpoint inhibitors such as nivolumab and pembrolizumab. To overcome TME-mediated resistance in both differentiated and undifferentiated thyroid malignancies, it is crucial to comprehend these interactions to optimize immunotherapeutic tactics, forecast treatment results, and create combination treatments.^[71]^

By increasing therapeutic efficacy and reducing off-target effects, targeted nanomedicine presents a viable approach to TC treatment. While cell-specific targeting makes use of ligands or biomimetic coatings to enhance cellular uptake, tumor tissue-targeting takes advantage of the EPR effect. To overcome resistance, organelle-targeted delivery routes carry medications to subcellular locations. Although clinical translation is still difficult because of the intricate targeting requirements, recent stimuli-responsive nanocarriers maximize therapeutic effects by integrating multistage tissue-, cell-, and organelle-targeting.^[21]^ The TME of pancreatic ductal adenocarcinoma is thick and complicated, which significantly reduces the effectiveness of nanodrug delivery systems (NDDSs). TME is characterized by an abundance of fibrotic stroma, which includes fibroblasts, immune cells, pericytes, and extracellular matrix components (collagen, HA, and fibronectin) in the presence of biophysical factors such as low oxygen levels, pH acidity, and high interstitial pressure. These barriers decrease drug concentration, hinder NP extravasation, and reduce tumor permeability. An additional reduction in the efficacy of nanotherapy is the immunosuppressive milieu of pancreatic ductal adenocarcinoma, which is characterized by low levels of cytotoxic T cells and natural killer cells, high levels of tumor-associated macrophages, cancer-associated fibroblasts, and regulatory immune cells. To overcome these challenges and improve therapeutic outcomes, adjusting nanocarriers to TME properties such as pH and enzymes is considered important.^[72]^ The TME is composed of an extracellular matrix, stromal cells, immune cells, and cancer cells. TME is essential for tumor proliferation, metastasis, immune evasion, and therapy resistance. By enhancing targeted drug delivery, regulated and stimuli-responsive release, and the regulation of immunosuppressive components inside the TME, nanocarriers will provide a promising strategy to deal with these challenges. New nanocarriers can enhance treatment efficacy and reduce recurrence by improving drug concentration, enhancing tumor permeability, stimulating immune effector cells, and decreasing off-target cytotoxicity. TME-targeted nanomedicine is a vital approach for enhancing the effectiveness of cancer treatment.^[73]^

### 4.2. Passive targeting mechanisms: enhanced permeability and retention effect^[22,74]^

Certain factors influence the size of NPs, including the EPR effect, circulation, biodistribution, and tumor-targeting efficiency. A balance is necessary for optimal tumor accumulation: NPs must be both large enough to prevent quick renal clearance and prolong circulation and small enough to efficiently enter the tumor vascular system. Although NPs with a diameter of 100 to 400 nm were thought to be the best for passive targeting, new research indicates that NPs with a diameter of <30 nm penetrate tumors more deeply. Designing efficient, low-toxicity NP-based therapies thus requires an understanding of size-dependent pharmacokinetics and the EPR effect.^[75]^ Selective accumulation of NPs in tumors is made possible by this effect, which results from leaky tumor vasculature and compromised lymphatics. Numerous investigations since its discovery in the 1980s have confirmed that the tumor retention of macromolecules is reliant on both size and MW, making the EPR effect a fundamental component of anticancer treatments based on NPs. Although practical translation is still difficult, techniques to increase EPR – through pharmacological, physical, and vascular-modulating approaches – have been developed over recent decades to improve tumor-targeting and therapeutic efficacy.^[76]^

Through the EPR effect, the surface characteristics of NPs, such as their hydrophilicity and charge, have a significant impact on their biodistribution, circulation duration, and tumor accumulation. Polythylene glycol (PEG) and polyzwitterions are examples of hydrophilic coatings that increase circulation, yet may limit tumor retention because of decreased tissue contacts (the “PEG dilemma”). By interacting with anionic tissue components, smart, charge-responsive NPs that are neutral in circulation but turn cationic in acidic tumor microenvironments improve tumor penetration and retention. These surface-engineered NPs exhibit improved intratumoral distribution and represent a promising approach for successful EPR-based cancer treatment.^[77]^ There are several histological subtypes of TC, and differentiated forms (PTC and follicular thyroid carcinoma [FTC]) have a good prognosis, whereas ATC and medullary (MTC) forms have a worse prognosis because of treatment resistance and early metastases. Conventional treatments, such as kinase inhibitors, chemotherapy, RAI therapy, and surgery, are frequently limited by microenvironmental barriers and tumor heterogeneity. By combining diagnostic and therapeutic modalities into a single NP platform, nanotheranostics improves biodistribution, tumor penetration, and therapeutic efficacy while enabling targeted drug administration, imaging, and customized treatment. Nanocarriers help the shift to customized medicine by providing accuracy in the treatment of TC, especially in aggressive or radioiodine-resistant variants, by utilizing the EPR effect.^[78]^

### 4.3. Active targeting mechanisms: receptor-mediated targeting (e.g., epidermal growth factor receptor and thyroid-stimulating hormone receptor [TSHR])

Because it is selectively expressed on thyroid follicular cells, the TSHR is an important target in TC treatment. By improving the uptake of RAI or drug carriers based on NPs, TSHR enables the precise delivery of medicines, especially in differentiated thyroid tumors (PTC and FTC). When applied to poorly differentiated or radioiodine-resistant cancers, TSHR-targeted strategies can increase specificity, reduce off-target damage, and overcome the drawbacks of traditional treatments. Personalized treatment plans and improved therapeutic efficacy in the management of TC are made possible by integration with nanotheranostic platforms, which further permit simultaneous imaging and therapy.^[79]^

### 4.4. Microenvironment-responsive drug release mechanisms (pH, reactive oxygen species [ROS], and enzymatic activity)

One intriguing approach to targeted cancer treatment is the use of pH-responsive NPs. In the acidic tumor microenvironment (pH < 6.5), these nanomaterials undergo structural or chemical changes that cause the regulated release of anticancer medications, yet they remain stable in blood and normal tissues (pH ~7.4). These methods increase drug accumulation at tumor locations, decrease off-target toxicity, and boost treatment efficacy by taking advantage of the pH gradient that exists between tumors and healthy tissues. For exact and effective treatment of tumors, many approaches, such as polymeric micelles, liposomes, dendrimers, inorganic NPs, and hybrid composites, have been developed to allow personalized drug loading and release.^[80]^ Because of the sensitivity of NPs to ROS, they provide direct targeted treatment by elevating the number of ROS in tumor microenvironments. These nanomaterials remain stable in healthy tissues, but if a high level of ROS exists, healthy tissues will alter structurally or chemically, thus causing anticancer medications to leak in a regulated manner. ROS-responsive systems increase medication accumulation at the tumor site, reduce off-target toxicity, and boost treatment efficacy by taking advantage of the oxidative stress feature of tumors. Numerous platforms, such as polymers based on sulfur, selenium, tellurium, and borate, provide accurate, stimuli-triggered drug delivery, providing a flexible strategy for safe and efficient cancer treatment.^[81]^

Recent reviews have fully discussed advances and challenges in TME and responsive nanomedicine, emphasizing the dynamic interplay of pH, ROS, and enzyme-triggered mechanisms in regulating drug release.

## 5. Therapeutic applications of ChNPs in TC

### 5.1. Preclinical in vitro studies on TC cell lines^[82,83,84]^

The difficulties of traditional chemotherapy, namely drug resistance mechanisms such as decreased uptake, increased efflux, and compromised apoptosis, are highlighted by in vitro research on TC cells. Because of these resistance routes, DOX, a commonly used medication in chemotherapy, has poor efficacy. By improving intracellular accumulation, permitting tailored distribution, and facilitating co-delivery with other therapies, chitosan-based nanostructures present a viable way around this restriction. Because of its naturally occurring, biological compatibility and ability to undergo redox and pH-sensitive changes, using chitosan-based nanostructures is a useful strategy for the administration of DOX in TC therapy.^[85]^ Hydrogel-based DDSs have been tested for their ability to release chemotherapeutics in a regular and sustainable manner in in vivo experiments on TC lines. In mice xenograft lines and by using glycol (GC) hydrogels, which had been photocured with visible light, water-soluble DOX-HCl was delivered directly into tumor tissue. GC-based hydrogels are a promising way to target DDSs for the treatment of TC by their ability to provide efficient localized administration, increased bioavailability, and improved anticancer activity.^[86]^

### 5.2. In vivo studies in thyroid cancer animal models

Exposure to blue light irradiation can generate cross-linked GC10/DOX hydrogel, which is formed by glycol chitosan, glycidyl methacrylate, and riboflavin, used as a photoinitiator. This results in a stable hydrogel with advantageous mechanical and swelling characteristics. DOX-HCl has been shown to release continuously over a number of days in in vitro experiments, and a porous shape appropriate for drug loading has been verified by field emision-SEM analysis. In comparison to controlled hydrogels, GC10/DOX has demonstrated effective cytotoxicity in cell viability tests employing FTC-133 TC cells. Comparing subcutaneous delivery of GC10/DOX near tumor sites to free DOX, in vivo evaluation utilizing FTC-133 xenograft mouse models has demonstrated a significant inhibition of tumor growth.^[87]^ The GC10/DOX hydrogel was used to assess the impact of DOX-loaded NPs on TC. Due to loosely interconnected methacrylated glycol chitosan chains created after 10-second visible light irradiation, the hydrogel swelled quickly, reaching equilibrium at 37°C in 5 hours with a swelling ratio roughly 8 times larger than its starting state. Due to drug diffusion from the inflated hydrogel matrix, in vitro release experiments have demonstrated an initial burst release of DOX-HCl within 18 hours, followed by continuous release over 7 days. Without the use of DOX-HCl, field emision-SEM examination of freeze-dried samples has shown uneven, porous surface and cross-sectional morphologies with interconnectivity, confirming its viability as a controlled drug delivery platform for the treatment of TC.^[88]^

### 5.3. ChNPs for chemotherapeutic delivery (e.g., DOX and anlotinib)

Chemotherapeutics such as DOX and anlotinib can be effectively transported by ChNPs due to their biocompatibility, biodegradability, and modifiability. They promote the internalization of DOX in cancer cells, prevent drug resistance by blocking P-glycoprotein, and enable synergistic therapy by co-delivery with additional anticancer drugs or nucleic acids. With the ability to be tailored for pH-, redox-, or light-responsive drug release, ChNPs provide a highly promising and effective cancer treatment.^[27]^ One of the main causes of cancer-related fatalities globally is non-small cell lung cancer. The limitations of the epidermal growth factor receptor inhibitor erlotinib include chemoresistance, low bioavailability, and poor solubility. TPGS-stabilized, erlotinib–chitosan-coated lipid NPs were created to address these problems. Through mucoadhesion, these NPs enhance oral absorption, prevent drug efflux, allow tumor-targeted administration via the EPR effect, and offer controlled release. The formulation’s intestinal penetration, stability, dissolution, and cytotoxicity against lung cancer cells have all been assessed and characterized.^[88]^

### 5.4. Synergistic and combination therapy using ChNPs

Chitin is a natural polysaccharide that has special biomedical implementations regarding toxicity, biodegradability, and biocompatibility. DDSs aim to increase therapeutic efficacy and reduce side effects through modifications that can be applied to the chemical structure. Conventional therapy, such as chemotherapy, radiotherapy, or targeted therapies, can be used, via chitosan-based NPs, to transport them precisely into the cancerous tissue. By overcoming issues such as toxicity, drug resistance, and bioavailability, this method can enhance the physiochemical and functional characteristics of cancer treatments.^[89]^ ChNPs, which are biocompatible carriers, can co-deliver several anticancer medications, including metformin (Met) and DOX, which can improve therapeutic efficacy. This combination is a significant approach for cancer treatment since it selects and targets many cellular pathways, enhancing drug concentration and decreasing resistance and side effects.^[90]^

## 6. Comparative analysis related to other polymeric nanocarriers

### 6.1. Bovine serum albumin-based NPs versus chitosan

Both chitosan and BSA influence calcium phosphate precipitation as they prevent the formation of crystalline phases by preventing amorphous calcium phosphate from transforming. However, because of its reliable molecular structure, chitosan exhibits a stronger inhibitory effect. The presence of these biopolymers influences the crystalline and amorphous phases’ structure and composition. This type of regulation allows precise adjustment of precipitates’ physicochemical characteristics, increasing the flexibility of approach to create multifunctional composites that may be used in regenerative therapy and bone tissue engineering.^[91]^ In this practical approach to the treatment of glaucoma, chitosan-coated bovine serum albumin NPs (CS–BSA–NPs) can be used by enhancing the ocular transport of tetrandrine. The chitosan coating improves transcorneal medication penetration, prolongs ocular residency, and strengthens mucoadhesion. Optimized CS–BSA–NPs (~238 nm, +24 mV) produce sustained release and high drug encapsulation (>95%). Biocompatibility increases cellular absorption, and higher antioxidant and antiproliferative activities have all been demonstrated in vitro. Evaluations conducted both in vivo and ex vivo have demonstrated a longer duration of intraocular pressure reduction and greater bioavailability than free tetrandrine. Given the circumstances, CS–BSA–NPs are considered to be an efficient nanocarrier strategy for ocular drug administration by integrating the advantages of both chitosan and albumin.^[91]^

### 6.2. Comparison to other polymeric carriers

Comparing ChNPs to other polymeric carriers, such as PLGA and PEG-modified systems, reveals clear advantages. While PEG has “stealth” properties that reduce phagocytic cell recognition and increase release time, chitosan also improves mucoadhesion and enhances absorption. PEG coatings reduce cellular uptake by both epithelial and immune cells, but in chitosan-coated carriers, this has not happened when related to experiments that compared PLGA and lipid NPs. These variations reflect the need to customize the polymer and surface alterations to the intended distribution method and therapeutic objective. PLGA and PEG are superior in stability and circulation lifetime, while chitosan offers a flexible strategy with highly improved bioadhesion and focused cellular interaction, making each system suitable for drug delivery applications. PLGAs have a low number of functional groups on the surface, which limits their ability to target and conjugate with active substances. By combining chitosan with PLGAs via chemical binding or physical adsorption, the surface charge can be reversed from negative to positive, thereby increasing the affinity for cancer cells and making a range of amine groups available for chemical reactions. Successful functionalization has been proved by zeta potential, X-ray photoelectron spectroscopy, and fourier-transform infrared spectroscopy characterization. With an initial burst followed by long-release administration, chitosan-modified PLGAs have also shown enhanced drug sustainability, improving cumulative drug release and therapeutic efficacy and preserving biological compatibility and degradability.^[92]^

### 6.3. Physiochemical and functional properties of ChNPs

ChNPs improve bioavailability, especially for insoluble medicines, by increasing drug EE up to 90% and prolonging release characteristics. Compared to conventional therapeutics, ChNPs are sensitive to pH; as a result, they improve absorption from 2 to 3 times by enhancing gelation in acidic media and modulating release in the colon. According to histological studies, ChNPs have minimal systemic toxicity, good biological compatibility, and reduced immunogenicity, which make them suitable for long-term use. Collectively, these characteristics enhance delivery of medication, improving patient adherence and clinical outcomes.^[93]^

### 6.4. Cost-effectiveness, scalability, and industrial translation potential of chitosan

ChNPs have a positive impact on DDSs, enhancing high encapsulation, achieving up to 90% capacity effectiveness, and providing sustained release, thereby improving biological availability, particularly for indissoluble medicines. The acidic environments and their release in target sites under controlled settings can be used to stabilize medications because they are pH-sensitive. ChNPs offer several advantages, such as minimizing systemic toxicity, enhancing biocompatibility, improving absorption 2 to 3 times more than conventional formulations (which makes them appropriate for long-term use), increasing selectivity and targeting, enhancing drug delivery, and improving clinical outcomes.^[92]^ Chitosan is an aminopolysaccharide, which is produced naturally from chitin and is used as an antibacterial and antifungal medication. It has applications in many fields, such as food, medicine, and agriculture. ChNPs have many characteristics, such as mucoadhesion, biodegradability, adhesion, solubility, and bioactivity. All of these characteristics can be affected by MW and the DD. Because of their antibacterial features, which are generated from electrostatic interactions that break down germ barriers, ChNPs have been used in curing wounds, drug delivery, dental care, food preservation, and water purification. These special characteristics make ChNPs a reliable approach to be applied in drug delivery, focused interactions, and long-lasting therapeutic outcomes.^[94]^

## 7. Clinical and translational challenges

### 7.1. Regulatory barriers and toxicity profiles in nanomedicine

Nanotechnology-enabled health products produced through nanotechnology can support medical diagnosis and treatment. The properties of these nanomaterials include being 1 to 100 nm in size and having several applications in the healthcare sector. This is due to their physicochemical features. Based on the number of nanoscale dimensions, they are divided into 3 classes: 1-dimensional (nanosheets and nanolayers), 2-dimensional (nanorods, nanotubes, and nanofibers), and 3-dimensional (NPs, quantum dots, and fullerenes). Despite their potential, some challenges have arisen in clinical translation in places like the US and the EU. It is important to understand the regulatory media and the toxicity associated with nanomedicine when they are integrated into healthcare to ensure their safety and efficacy.^[92]^ NPs have many special characteristics and interactions, but they should be tested to avoid any serious regulatory and safety issues for gene vaccines and nanomedicine. Guidelines are used to assess standards related to safety, ethics, and the balance between public use and intellectual property. One example of this is the lipid NP, which was used in COVID-19 vaccines and which overcame multiple obstacles around the world. These regulations are important in the control of the use of nanomedicine in improving global healthcare.^[95]^

### 7.2. Limitations in large-scale manufacturing, repeatability, and the stability of NPs

The stability, safety, and reproducibility of NP production at the manufacturing scale present notable limitations. Although there are many synthesis methods, consistency and material efficiency are usually compromised when scaling up from the lab to industrial manufacture. Although improvements in posttreatment modifications and synthetic procedures are intended to improve the quality of NPs, overcoming these obstacles is essential for the long-term and reliable use of nanomaterials in electronics, medicine, and other fields.^[96]^ Innovative nanomedicine applications have been made possible by the development of nanotechnology, whereby the therapeutic potential of NPs is determined by their size, surface features, drug loading, targeting ability, and multifunctionality. Although polymeric NPs (PNPs) show great promise, repeatability, homogeneity, and property control are issues in large-scale manufacture. Upscaling is necessary yet difficult since traditional laboratory synthesis techniques are prone to batch-to-batch variability. Recent advancements have focused on creating quick, controllable, and repeatable high-productivity techniques for PNP synthesis that take advantage of biocompatibility and biodegradability. Since PNP industrial production faces comparable difficulties, insights from metal NP upscaling offer a helpful direction.^[58]^

### 7.3. Immunogenicity, biocompatibility, and long-term safety of chitosan NPs

The reduction of immunogenic reactions, biocompatibility, and long-term safety – all of which are essential for clinical translation – remain difficult to achieve despite their promise. Safety cannot be assumed across dose ranges or formulations, despite the biocompatibility that is generally attributed to ChNPs, as evidenced by previous in vivo toxicity evaluation literature. In 1 study, glycol ChNPs were evaluated for systemic toxicity in healthy mice at repeated high doses. The severity of the inflammatory responses, cardiotoxicity, tissue damage, and organ dysfunction increased with the number of doses used.^[97]^ Furthermore, certain ChNPs such as chitosan/poly(γ-glutamic acid) NPs were demonstrated to shift human antigen-presenting cells toward a pro-inflammatory phenotype, illustrating that ChNP–immune interactions can be formulation-dependent.^[98]^

### 7.4. Human clinical trial challenges and lack of information about TC

Despite conventional therapies, such as surgery, radiotherapy, and chemotherapy, most endocrine cancers, including TC, have a poor prognosis. To overcome these results, multi-treatments combining, for example, immunotherapy, chemoradiotherapy, and multi-targeted tyrosine kinase inhibitors, have been suggested. In customized medical methods, NDDS can ease the process by enhancing targeted therapy, using imaging-assisted surgery, and reaching a diagnosis. However, there are a small number of human clinical trials for TC, and there is a dearth of reliable data about the long-term results, effectiveness, and safety of NDDS in TC. This disparity highlights the need for urgent clinical research to prove the role of nanotechnology as a promising approach to enhancing TC management.^[69]^

### 7.5. Individualized nanomedicine in TC therapy

Nanomedicine offers a potential method for customized TC treatment by tailoring medicines to each patient depending on immunological characteristics and the genetic makeup of the tumor. By using targeted DDSs, treatment efficacy is increased, and side effects are decreased. Current developments include authorized nanopharmaceuticals and nanoformulations in clinical trials, as well as sophisticated instruments like clustered regularly interspaced short palindromic repeats/Cas9 genome editing and organ-on-a-chip models for precise diagnosis and treatment. Despite challenges with large-scale production, ethics, safety, and cost, customized nanomedicine has the potential to revolutionize the treatment of TC by integrating precision oncology, early diagnosis, and patient-specific immunotherapy.^[99]^

## 8. Future directions and research opportunities

### 8.1. Integration with smart and responsive platforms

To address medication resistance and reduce side effects, hydrogels, which are 3D polymeric networks, provide a flexible platform for the targeted and sustained delivery of chemotherapeutics like DOX. While co-delivery with genes or other medications enables polychemotherapy and better tumor suppression, natural-based hydrogels (such as chitosan, alginate, and gelatin) and stimuli-responsive systems (pH, redox-, and thermosensitive medications) improve drug internalization and cytotoxicity. The addition of NPs greatly increases intracellular uptake and stabilizes medication release. Combining hydrogels with intelligent nanostructures offers a viable way to circumvent chemoresistance and improve the results of cancer treatment.^[100]^ A flexible platform for targeted chemotherapy, nanogels are hydrogel-based NPs (1 nm–1 μm) with a high drug-loading capacity, adjustable shape, and biocompatibility. Their adaptable structure protects therapeutic materials, including macromolecules and small compounds, increases tumor penetration, and extends circulation. Targeted and controlled distribution is made possible by functional groups (carboxyl, disulfide, hydroxyl, and amino) that facilitate precise drug conjugation, cross-linking, and stimuli-responsive release (pH, redox, temperature, and light). Both synthetic and natural polymers are used, and a variety of preparation techniques are used to improve stability and effectiveness. Nanogels enhance therapeutic results, lower toxicity, and offer a viable method for tailored cancer treatment by combining controlled release with multifunctional design.^[101]^

### 8.2. Development of theranostic systems for combined diagnosis and therapy

An important improvement in cancer treatment is the creation of multifunctional theranostic NPs that combine diagnosis and therapy. An example of an NP is Bi_2_O_3_/CS@5-aminolevulinic acid–curcumin. Approximately 80 nm in size, this NP is produced by sol-gel techniques and has been assessed for structural stability and performance. The mechanism of action of such NPs is to enhance radiosensitization up to 450%, elevate the production of ROS, and target the cytotoxicity effect on SKBr-3 cancer cells while bypassing healthy cells. Then, CT imaging is used to view and direct the radiation into the tumor. As in vivo studies have demonstrated, Bi_2_O_3_/CS@5-aminolevulinic acid–curcumin NPs have shown a significant reduction in tumor size when low-dose radiation and NP administration were combined.^[102]^ Real-time monitoring and individualized cancer therapy are integrated into therapeutic and imaging chemicals in a single carrier, which is called a theranostic system. Polysaccharides offer modifiable, compatible, and degradable matrixes in biological systems for the co-administration of contrast agents and medications. Recent advancements that aim to enhance the effectiveness, safety, and precision of cancer treatment focus on the potential of multimodal imaging like magnetic resonance imaging, computed tomography, positron emission tomography, single-photon emission computed tomography, ultrasound, and toxicity reduction.^[103]^

### 8.3. Artificial intelligence (AI)-aided NP design for optimized drug delivery

Recently, AI has been introduced as an advanced technology that can be used for perfecting drug delivery using NPs when combined with NPs. For example, a lecithin/chitosan combination profile was used as a model drug, which is weakly soluble in water, to enhance NP size, polydispersity, efficiency, and the silymarin release profile. AI models have been used to predict drug release dynamics, particularly for formulations that are 161 nm in size, have 97% efficacy, and a zeta potential of + 38 mV. The improved NPs’ increased cytotoxicity against cancer cells shows how AI-driven design may replace empirical approaches in pharmaceutical development with data-based precision.^[104]^ AI and machine learning make drug delivery more precise, effective, and customized when they are combined, and they have been seen as a therapeutic development. These technologies assess the biological and chemical information of the NP carriers to optimize stability, bioavailability, and tissue-targeting. AI-driven models have advantages related to time, cost, and risk, in addition to the control of medication release and the evaluation of compliance with standards. AI and machine learning present a new technology to make therapy more effective and personalized by fusing computer intelligence with nanotechnology. This opens an opportunity for data-driven precision medicine to replace certain conventional medicines.^[105]^

### 8.4. Expansion of chitosan-based nanomedicine to other endocrine and solid tumors

ChNPs have helped to increase the safety, selectivity, and bioavailability of anticancer medications for several types of cancer, ranging from solid to endocrine malignancies. By overcoming the physiological limitations of conventional medicines, NPs can control the pharmacokinetic parameters of the drug. Preclinical and clinical research into incorporating chemotherapeutics, such as paclitaxel and docetaxel, into ChNPs has confirmed an increase in drug release and efficacy at each biological level, in addition to reductions in toxicity. Chitosan nanocarriers offer a promising model that aims to enhance treatment outcomes in diverse cancer types, such as lung, colorectal, breast, and prostate carcinoma. This is due to their high selectivity and ability to differentiate between malignant and healthy cells, highlighting their potential role as a broad-spectrum approach in advanced cancer therapy.^[106]^ Since cancerous cells often become resistant to conventional treatments such as chemotherapy and radiotherapy, the introduction of new techniques is vital to enhance the management of endocrine cancer.

## 9. Challenges and a future perspective for the clinical translation of ChNPs

Although ChNPs have shown great promise in preclinical TC models, a number of obstacles remain to their effective use in clinical settings. The absence of standardized large-scale production techniques that can generate NPs with similar physicochemical characteristics, such as particle size, zeta potential, drug-loading capacity, and release kinetics, is a significant drawback. Variability across batches can have a significant impact on regulatory approval, repeatability, and therapeutic efficacy.

Another significant barrier to clinical deployment is regulatory concerns. To guarantee product efficacy and consistency, nanomedicine requires thorough characterization, quality control testing, and safety assessment. Additionally, compared to traditional pharmaceutical formulations, the regulatory frameworks for NP-based treatments are less developed, which could lead to the postponement of clinical approval and commercialization.

Another crucial issue is long-term safety. Despite the widespread belief that chitosan is friendly and biodegradable, nothing is known about the effects of long-term biodistribution, metabolism, immunogenicity, and possible storage of ChNPs in human tissues. While there is still a dearth of clinical data, the majority of available information comes from in vitro and animal investigations.

Standardized manufacturing procedures, thorough toxicological and pharmacokinetic evaluation, targeted strategy optimization, and well-planned clinical trials should be the main topics of future study. Transforming the encouraging preclinical performance of chitosan-based nanocarriers into commercially useful treatments for TC will require these issues to be addressed.

## 10. Conclusions

Conventional TC therapies remain limited by systemic toxicity, poor drug selectivity, and challenges associated with the tumor microenvironment. ChNPs have shown promise as DDSs that can lower off-target side effects, improve tumor-specific targeting, and increase drug bioavailability. Preclinical research indicated that through improved cellular uptake and regulated drug release, ChNPs may boost the delivery and therapeutic efficacy of anticancer drugs. Additionally, surface-engineering techniques, including ligand conjugation, PEGylation, and stimuli-responsive alterations, have shown promise in enhancing TC targeting effectiveness. Despite promising outcomes, a number of obstacles still prevent clinical translation, including batch-to-batch reproducibility, manufacturing scalability, regulatory approval requirements, and the absence of thorough long-term safety and pharmacokinetic data in humans. These difficulties demonstrate the need for additional translational research, consistent evaluation techniques, and carefully planned clinical trials. Future studies on chitosan-based formulations should focus on translating these observations into clinical settings, targeting thyroid-specific receptors, increasing ligand selection, and evaluating the safety and pharmacokinetics parameters. Moreover, all related sciences, such as pharmacology and oncology, are critical in the transformation of chitosan-based nanocarriers from an experimental approach into a real treatment option to manage TC.

## Author contributions

**Conceptualization:** Hamad S. Alyami.

**Data curation:** Hamad S. Alyami, Nora A. Ahmadi.

**Formal analysis:** Hamad S. Alyami.

**Funding acquisition:** Hamad S. Alyami.

**Investigation:** Hamad S. Alyami, Nora A. Ahmadi.

**Methodology:** Hamad S. Alyami.

**Project administration:** Hamad S. Alyami.

**Resources:** Hamad S. Alyami.

**Software:** Hamad S. Alyami.

**Supervision:** Hamad S. Alyami.

**Validation:** Hamad S. Alyami.

**Visualization:** Hamad S. Alyami.

**Writing – original draft:** Hamad S. Alyami, Nora A. Ahmadi.

**Writing – review & editing:** Hamad S. Alyami, Nora A. Ahmadi.
